# Adherence to Swedish national pregnancy dating guidelines and management of discrepancies between pregnancy dating methods: a survey study

**DOI:** 10.1186/s12978-019-0760-3

**Published:** 2019-07-04

**Authors:** Merit Kullinger, Michaela Granfors, Helle Kieler, Alkistis Skalkidou

**Affiliations:** 1 Department of Women’s and Children’s Health, Uppsala University, Uppsala, Sweden; 2 Region Vastmanland – Uppsala University, Center for Clinical Research, Hospital of Vastmanland Västerås, Kvinnokliniken, Västmanlands sjukhus, 721 89 Västerås, Sweden; 3 Department of Medicine, Solna, Clinical Epidemiology Unit, Karolinska Institutet, Stockholm, Sweden; 4 Department of Clinical Science, Karolinska Institutet, Danderyd Hospital, Stockholm, Sweden; 5 Department of Medicine, Solna, Centre for Pharmacoepidemiology, Karolinska Institutet, Stockholm, Sweden

**Keywords:** Pregnancy, Gestational age, Estimated day of delivery, Ultrasound, Last menstrual period, Clinical practice, Adherence to guidelines, Pregnancy dating

## Abstract

**Background:**

Swedish national guidelines for pregnancy dating were published in 2010. Follow-up is needed to assess adherence and to identify whether any clinical topics are not covered in the guidelines.

**Methods:**

All units in Sweden that performed ultrasound-based pregnancy dating were asked to complete a web-based questionnaire comprising multiple-response questions and commentary fields. Information was collected regarding baseline information, current and previous clinical practice, and management of discrepancies between last-menstrual-period- and ultrasound-based methods for pregnancy dating.

**Results:**

The response rate was 79%. Half of the units offered first-trimester ultrasound to all pregnant women. However, contrary to the guidelines, the crown–rump length was not used for ultrasound-based pregnancy dating in most units. Instead, ultrasound-based pregnancy dating was performed only if the biparietal diameter was between 21 and 55 mm. Management of discrepancies between methods for pregnancy dating varied widely.

**Conclusions:**

The units reported high adherence to national guidelines, except for early pregnancy dating, for which many units followed unwritten or informal guidelines. The management of discrepancies between last-menstrual-period-based and ultrasound-based estimated day of delivery varied widely. These findings emphasize the need for regular updating of national written guidelines and efforts to improve their implementation in all units.

## Plain English summary

National Swedish guidelines were published in 2010 regarding how pregnancy length should be estimated by the use of ultrasound. This study is based on a survey that was sent in 2017 to all units that perform ultrasound examinations to estimate pregnancy length in Sweden. The aim was to describe the units’ present and past routines in relation to the national guidelines. Also, there were questions on what the units did if there was a difference between the pregnancy length when calculated from the last menstrual period or estimated by the fetus’s size at an ultrasound examination. For most parts, the units followed the guidelines, with the exception of early ultrasound examinations. In many units fetal measurements corresponding to a pregnancy length of 11 weeks were not used to estimate a pregnancy length and the results from an early ultrasound examination were only used if the measurements corresponded to a pregnancy length of at least 12 weeks. When there were two different pregnancy lengths if last menstrual period or if ultrasound was used, some units planned a follow-up and some did not. These results stress the importance of following up the implementation of new guidelines to perceive which routines are actually used and if there is a need to renew the guidelines.

## Key message

Except for early dating, adherence to the Swedish national guidelines on pregnancy dating was good. Management of discrepancies between menstrual-period-based and ultrasound-based gestational age varied widely.

## Background

Estimating gestational age correctly is fundamental to provide high quality antenatal care. With the introduction of obstetric ultrasound, there has been a shift from last-menstrual-period-based to ultrasound-based estimation of gestational age. The method and the timing of ultrasound pregnancy dating is important because it can affect the precision of pregnancy dating, rates of pre- and postterm birth, and rates of small-for-gestational-age infants [1–3]. There are national and international guidelines on pregnancy dating. However, national guidelines are not always implemented as intended, and follow-up is needed to assess adherence [4, 5].

Pregnancy dating by ultrasound was introduced in Sweden during more than a decade, starting in 1976 [6]. A 1996 survey based on answers from 55 of the 59 ultrasound units in Sweden reported that ultrasound pregnancy dating was applied by 52 units in the second trimester (at week 16–20) and three units in the first trimester (week 10–15) [6]. Second-trimester ultrasound examination for pregnancy dating and anomaly screening is now routinely offered to all pregnant women, free of charge, as part of the maternal health care program in Sweden, and is typically performed by a specialized midwife. First-trimester ultrasound examination has been introduced to a varying degree, mostly in the context of chromosomal screening [7].

According to Swedish law, the county councils are required to provide good-quality, need-based health care equally to all citizens [8]. When there is special need for prioritization and to help decision-making based on population needs, the Social Board of Welfare publishes national guidelines [9]. However, most guidelines are produced by the clinics themselves or by a medical specialty college. In 2010, a workshop committee installed by initiative of the ultrasound section of the Swedish Association for Obstetricians and Gynecologists published fetal biometry and pregnancy dating guidelines after one year of preparations. The guidelines were discussed at section meetings, as well as the association’s yearly meeting, and were then openly published on the association’s homepage [10]. The current Swedish as well as international guidelines recommend pregnancy dating based on a first-trimester ultrasound examination, if performed [10, 11]. The Swedish guidelines state that pregnancy dating can be performed between 11 and 22 weeks of gestational age, and preferably at 11–14 weeks, based on the crown–rump length (CRL) until the biparietal diameter (BPD) is 21–55 mm [10]. In the United States, for example, guidelines recommend pregnancy dating on ultrasound measurements only if there is a certain discrepancy with the last-menstrual-period-based estimate [12].

To our knowledge, no study since 1998 has described the Swedish clinical practice in pregnancy dating or adherence to guidelines. There is a lack of studies on clinical practices regarding the perceived reliance of estimated gestational age. Commonly, gestational age is first estimated according to the last menstrual period and then by ultrasound. There is often a discrepancy between these two methods, and larger discrepancies are associated with adverse perinatal outcomes [13]. Management of discrepancies between pregnancy dating methods are not included in the Swedish guidelines, and it is unknown how discrepancies are managed in clinical practice.

The primary aims of this study were to describe current practice for pregnancy dating in Sweden and to assess adherence to guidelines on pregnancy dating. The secondary aim was to assess clinical practice concerning discrepancies between methods for pregnancy dating.

## Material and methods

All units in Sweden that perform ultrasound examinations for pregnancy dating purposes were asked to complete a web-based questionnaire. Health care in Sweden is administered in 21 counties, and contact details for the ultrasound units were found through each county’s official webpage or through personal contacts.

The questionnaire included 30 question items divided into four dimensions: 1) baseline information about the responder and the unit; 2) the unit’s current clinical practice for pregnancy dating; 3) former practice and changes over time; and 4) assessment of the accuracy of the estimated gestational age and how discrepancies between the last-menstrual-period- and ultrasound-based methods were handled. Questions and replies were written in Swedish (a translated copy of the questionnaire is available on request). The questionnaire comprised mainly multiple-response questions but included commentary fields. The quoted comments have been translated into English.

The questionnaire was piloted among three fellow obstetricians and, after adjustments, was distributed from a web-based platform. After two reminders or in cases of invalid email address, the survey was sent by post.

### Statistical analyses

The replies were electronically registered or entered manually for replies by post. Analyses were conducted using descriptive statistical methods in IBM SPSS Statistics version 24.0.0.2. If the answers to multiple-response questions did not coincide with the answers in comments, priority was given to information stated in comments.

### Ethical approval

The Regional Ethical Review Board in Uppsala, Sweden, approved the study (reference number 2012/412, amendment approved November 15, 2017).

## Results

**Fig. 1:**
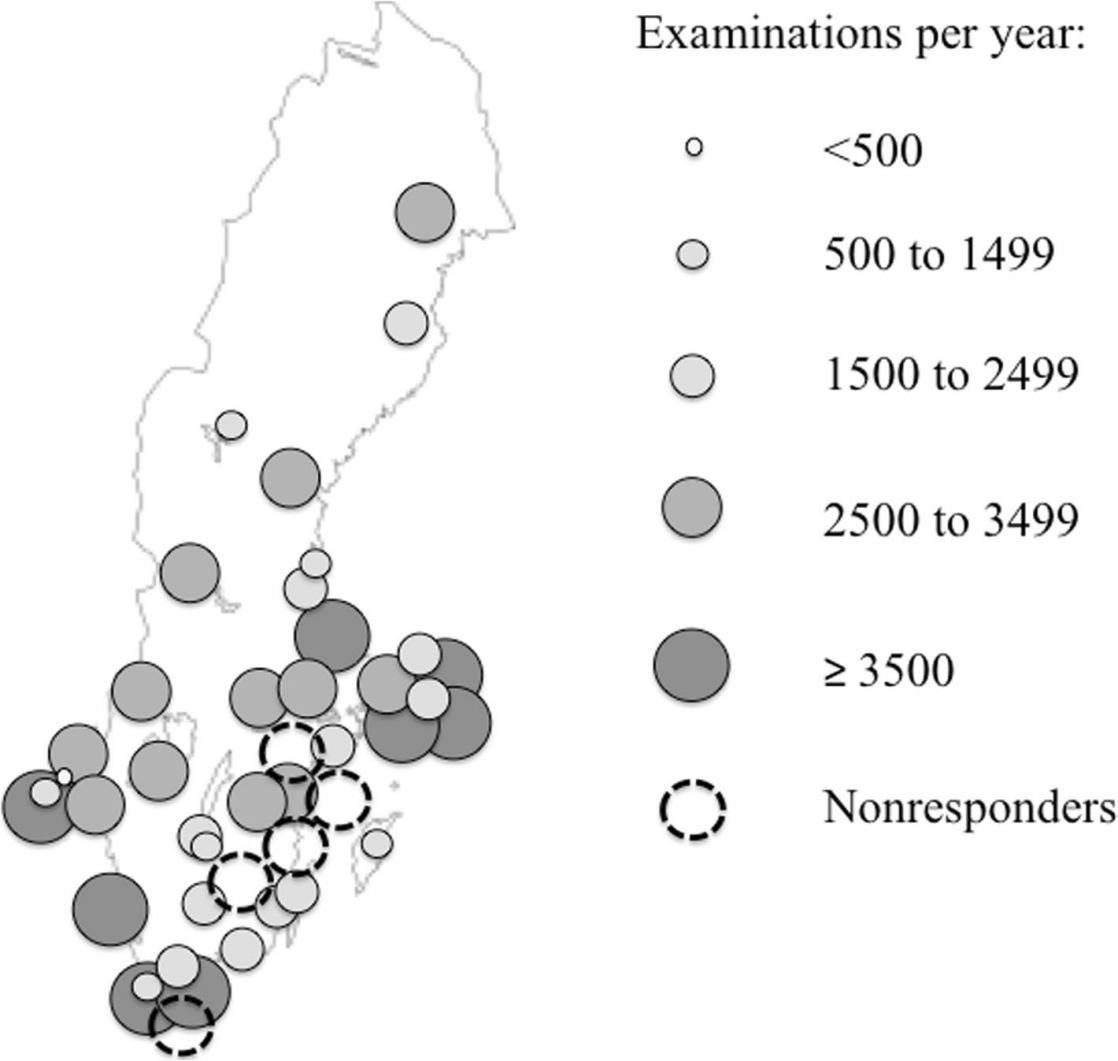
Responders’ (*n* = 38) replies about the number of second-trimester routine ultrasound examinations performed per year. Units with no reply (*n* = 5) are represented by dashed circles

The response rate was 79%: 38 valid replies, five nonresponses, and five excluded responses (two smaller units covered by larger units’ responses, two double answers, and one blank, anonymous answer). There was at least one reply per county (Fig. 1). Based on the reported minimum number of second-trimester ultrasound examinations in relation to births per county, the missing replies at most corresponded to 16% of total births [14].

Characteristics of responders and ultrasound units are presented in Table 1. There was a shift from pregnancy dating based on last menstrual period to second-trimester ultrasound between 1980 and 1992, based on answers from 19 units (there were no answers on this item from the remaining 19 units, of which some did not exist during this time period). First-trimester ultrasound examination was introduced as the primary method in one unit, at the university clinic in Linköping in 1983, where second-trimester ultrasound was added in 2007.

**Table 1:** Characteristics of responders by number (*n*) and percentage (%) of total ultrasound units (*n* = 38 units)

Characteristics	(*n*)	(%)
Position of responder		
Head of department	6	15.8
Physician in charge of ultrasound unit	21	55.3
Midwife in charge of ultrasound unit	6	15.8
Other specified position	4	10.5
Missing	1	2.6
Type of unit		
University hospital	6	15.8
Regional hospital	20	52.6
Private unit	6	15.8
Other or mixed	6	15.8
Second-trimester ultrasound examinations per year		
<500	1	2.6
500 to 1499	7	18.4
1500 to 2499	10	26.3
2500 to 3499	12	31.6
≥3500	8	21.1
First-trimester ultrasound examinations offered in unit^*a*^		
<11 weeks on woman’s request	3	7.9
<11 weeks on indication	7	18.4
Week 11 to 14 for all women	9	23.7
Week 11 to 14 only for specified groups in the context of chromosomal screening	16	42.1
Week 11 to 14 for all women in the context of chromosomal screening	16	42.1
BPD measurements for pregnancy dating in the unit		
BPD 21–55 mm	33	86.8
BPD 32–55 mm	2	5.3
BPD and FL combined	2	5.3
Missing	1	2.6
CRL measurements for pregnancy dating in the unit		
Never been practiced	13	34.2
Previously performed but currently only BPD-based	6	15.8
Limited to CRL ≥45 mm and BPD ≤21 mm	11	28.9
Limited to measurements of 45–85 mm	2	5.3
Other, such as occasional use	4	10.5
Missing	2	5.3
Gestational age at EDD		
39 weeks + 6 days	30	78.9
40 weeks + 0 days	5	13.2
Uncertain	2	5.3
Missing	1	2.6

*BPD* biparietal diameter, *CRL* crown–rump length, *EDD* estimated day of delivery, *FL* femur length

^*a*^More than one alternative could be marked

**Fig. 2:**
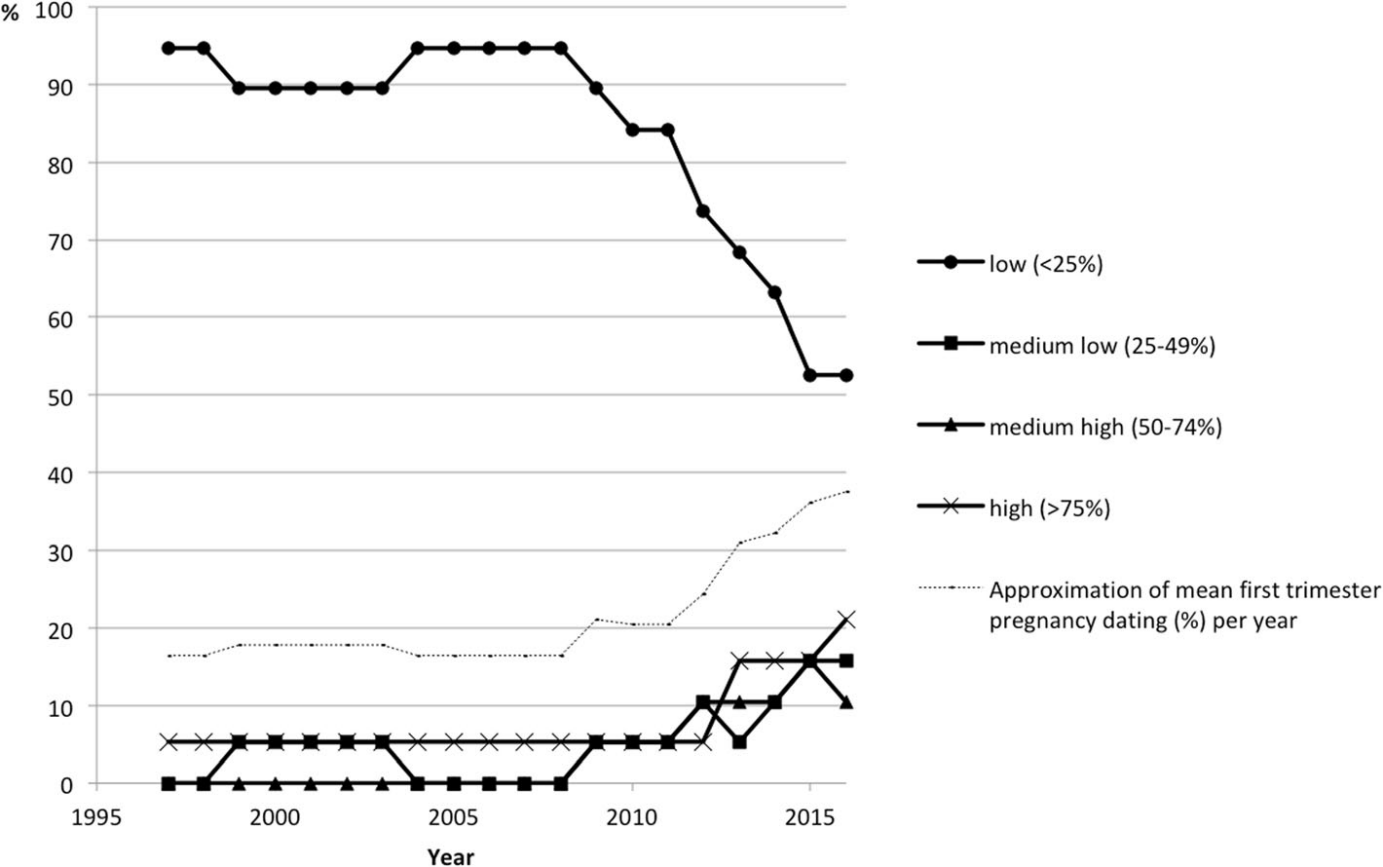
Estimated percentage of pregnancy dating ultrasound examinations performed in the first trimester instead of the second trimester during the years 1997–2016 based on the 19 answers covering the entire time. The thin line represents an approximation of the mean percentage of first-trimester pregnancy dating ultrasounds for each year, based on the median value for each category definition

In 2017, when the survey was performed, a first-trimester ultrasound examination was offered to all women in 19 of the 38 units, was offered to part of the women in 17 units, was not offered in one unit, and there was no answer from one unit. Between 1997 and 2016, the estimated percentage of ultrasound pregnancy dating performed in the first trimester instead of the second trimester increased (Fig. 2).

Thirty units applied a gestational length of 39 weeks + 6 days to estimate the date of delivery in connection with pregnancy dating and five units used 40 weeks + 0 days. Two units were uncertain on which gestational length was used (Table 1).

In 26 units (68%), for at least part of their population, pregnancy dating routines were changed from the second to first trimester 2008–2015. In 11 units (29%), pregnancy dating was preferably based on CRL or BPD at week 11–14, following guidelines [10]. In 21 units (55%), pregnancy dating was based on a first-trimester ultrasound only if the BPD was ≥21 mm (corresponding to 12 weeks + 3 days). In five units (13%), pregnancy dating was always based on second-trimester ultrasound examinations, although first-trimester measurements existed. One unit had missing answer. CRL-based pregnancy dating was commented: *“pregnancy dating by CRL [is performed] only occasionally (neutral fetal position, favorable circumstances)”* and *“We await recommendations … [our] experience is that CRL performs worse”*. In summary, in 26 units (68%), first-trimester ultrasound examinations between 11 weeks + 0 days and 12 weeks + 2 days were not used for pregnancy dating (Table 2).

**Table 2:** Adherence to national guidelines for pregnancy dating

National guidelines – recommendations for pregnancy dating	Unit follows guidelines		Unit does not follow guidelines		Missing information	
	(*n*)	(%)	(*n*)	(%)	(*n*)	(%)
First trimester-based (11–14 weeks), if performed	11	28.9	26	68.5	1	2.6
CRL-based, if 11 weeks + 0 days to 12 weeks + 2 days	11	28.9	24	63.2	3	7.9
BPD-based, if 12 weeks + 3 days to 22 weeks + 5 days	34	89.5	3	7.9	1	2.6
Based on size of largest fetus in multiple pregnancies	37	97.4	0	0.0	1	2.6
Based on days since embryo transfer + number of cultivation days + 14 days in ART pregnancies	30	78.9	1	2.6	7^*a*^	18.5

*CRL* crown–rump length, *BPD* biparietal diameter, *ART* artificial reproduction technique

^*a*^Three units were categorized as missing because the responder stated that the estimated date of delivery was provided by the ART clinic

The adherence to pregnancy dating recommendations was high for BPD measurements, multiple pregnancies, and artificial reproduction technique pregnancies (Table 2). Seven units had stopped using CRL-based pregnancy dating between 2014 and 2017, because of information provided at national meetings on the interpretation of the guidelines and an observed increase in postterm rates [15]. One unit commented that the change was made *“because [name of senior ultrasound expert] clarified that … recommendations are valid only for BPD measurements.”* Respondents commented that the units in Stockholm County had agreed to stop CRL-based dating because of an observed increase in postterm rates: *“We, in Stockholm, agreed on dating based on BPD ≥21 mm to do the same.”*

More responders would consider using an EDD based on a second-trimester rather than first-trimester ultrasound examination when examination was performed elsewhere.: *“...[the EDD] will be used if performed by a certified unit and preferably using the BPD. The CRL can be acceptable if coincident with the actual measurements”.* Another topic that emerged from the comments was the need for documentation in some situations such as *“unreliable examination abroad, or when documentation is inadequate”.*

National guidelines contain no recommendations for the management of discrepancies between methods. However, the survey questions asked about eventual follow-up in cases of a discrepancy. In 21 units (55%), a follow-up was scheduled if the ultrasound-based estimate was smaller than expected based on the last menstrual period by at least − 8 to − 14 days (median − 14 days). In six units (16%), a follow-up was scheduled if the estimate were larger than expected by at least + 12 to + 14 days (median + 14 days). The mean time to follow-up was 14 days (range 7–21 days).

In 26 units (68%), the clinical management (for example, date for labor induction) would not be affected by a difference in EDD based on the women’s self-report of the date of conception or the result of a positive ovulation test compared with the EDD estimated by ultrasound. However, in six units (16%), this information could influence clinical decisions.: *“…clear indications that the EDD could have been set at a date [that was] too late will lead to individual planning; for example, postterm control one week earlier.”* The responses from six units (16%) had missing answers. Comments included for example that in case of discrepancy the unit performed a *“repeated ultrasound examination to verify the EDD.”*

Several factors were reported to affect the assessment of the reliability of the ultrasound-based EDD (Table 3). Comments expressed diverse views. One unit wrote: *“An EDD based on ultrasound is considered valid in our clinic; this [that is, an assessment of the method’s precision] has never been discussed if a patient is dated according to guidelines…”*. By contrast, another unit commented: *“Everyone with a significant discrepancy is evaluated by a physician using ultrasound”*.

**Table 3:** Factors that could influence assessment of the ultrasound-based estimated date of delivery

	Number of units^*a*^	(%)
Large discrepancy between last menstrual period and ultrasound estimates	10	(3, 26)
Fetal sex	3	(7, 9)
Self-reported reliable date of conception or positive ovulation test	10	(3, 26)
Fetal malformations affecting fetal measurements	30	(78,9)
Discrepancy between ultrasound pregnancy dating and earlier CRL measurements	17	(44,7)
Discrepancy between ultrasound pregnancy dating and earlier BPD measurements	20	(52,6)
Several combined factors indicating that the estimated gestational age by ultrasound is less reliable	24	(63,2)

*CRL* crown–rump length, *BPD* biparietal diameter

^*a*^More than one answer could be reported per unit. In six of the 38 units, no answer was marked

## Discussion

The responders indicated overall good adherence to the national guidelines, with the exception of early pregnancy dating based on CRL measurements. Another finding was that the management of discrepancies between methods for pregnancy dating in clinical practice varied widely, probably because of the lack of recommendations for managing such discrepancies in the national guidelines [10].

Although many units offered a first-trimester ultrasound examination, surprisingly few units applied pregnancy dating based on that examination. The estimated proportions of first trimester pregnancy dating were similar in comparison with 2016 register-based estimates of first-trimester (36%; 7% on CRL and 29% on BPD) and second-trimester pregnancy dating (64%) [16]. In up to two-thirds of the units, the results of a first-trimester ultrasound examination would not be used for pregnancy dating purposes if the fetal BPD was < 21 mm. In one of 10 units, the results from any first-trimester ultrasound examination would be disregarded for pregnancy dating purposes. This is contrary to both Swedish and international guidelines, although some variation is expected, even in a small country, as is the case for other antenatal routines [4, 5, 10, 11]. However, the deviations from the guidelines were related to observed challenges after implementation, such as increased postterm rates that were attributed to the new dating formulae [15].

When first-trimester ultrasound results are disregarded, pregnancy dating would instead be based on second-trimester ultrasound examinations. There is a long tradition of performing mainly second-trimester ultrasound examinations in Sweden. This may explain the priority given to second-trimester pregnancy dating, which also applied when pregnancy dating had been performed in another unit. Another obstacle seemed to be the lack of reliable documentation when pregnancy dating had been performed in another county or country. Additionally, units that have not yet implemented first-trimester ultrasound examinations for chromosomal screening have less training and could be more reluctant to perform CRL-based pregnancy dating.

The predominant practice of disregarding CRL measurements, and the associated comments, indicated the units’ adherence to unwritten or informal recommendations. One reason for the use of these unwritten or informal recommendations was the observed increase in postterm rates with CRL-based pregnancy dating, which has been noted earlier, when pregnancy length is not calibrated to the same median [15, 17]. Although first trimester CRL measurements generally are more precise for pregnancy dating than second trimester measurements, the reported increase in post term rates after introducing CRL measurements for pregnancy dating could be due to problems with the used formulae or the definition of pregnancy length [15]. A tendency to be strongly influenced by informal pathways has already been studied in the obstetric setting [4]. However, it seemed like this practice had changed in only some counties, and almost one-third of the units still followed the written guidelines.

By contrast, adherence to specific recommendations for pregnancy dating was very high—for example in multiple or assisted reproductive therapy pregnancies. The responders made no comments indicating that the guidelines were insufficient or difficult to interpret. In general, adherence was higher than expected when compared with other studies of adherence to national guidelines [4, 5].

In many units, a follow-up would be planned if the gestational age estimated by ultrasound was 2 weeks shorter than that estimated from the last menstrual period. The vast majority also reported that in cases of a discrepancy between menstrual-period- and ultrasound-based gestational age, a new ultrasound examination would be planned after 14 days, although this is not included in the national guidelines [10]. This may be the correct approach for assessing early growth deviations, although later growth deviations would not be addressed by this practice [13, 18, 19]. When the fetus is smaller than expected based on the last menstrual period, follow-up may be motivated by the increased risk of adverse neonatal outcomes, such as intrauterine or neonatal death [13, 18, 19]. None of the units mentioned fetal weight estimation later during pregnancy, despite the increased risk of being small for gestational age at birth in this group [20].

Discrepancies between the EDD by the last menstrual period and by ultrasound are common [21], and women’s additional information on a plausible date of conception may contradict the ultrasound-based estimated gestational age. In Sweden, clinical decisions will usually be based only on the ultrasound estimate. In some other countries, ultrasound is used for pregnancy dating only in the case of a defined discrepancy of at least 5 or 7 days between the EDD by last menstrual period and ultrasound [12].

We found that the units used a variety of ways to manage discrepancies between last-menstrual-period-based and ultrasound-based gestational age; this may also occur in other similar settings. Interestingly, neither national nor international guidelines mention such a discrepancy to be a risk indicator [10, 11], despite associated risks for both mother and infant, such as preeclampsia or low birthweight [13, 18, 19]. Discrepancies between methods for pregnancy dating and the suggested follow-up may need to be considered in national and international guidelines, regardless of which method is given priority for determining the EDD. Some issues to be addressed are the discrepancy threshold for follow-up and the type of follow-up that should be recommended.

The strengths of our study include the high response rate and the representation of all counties among responders: more than 84% of the pregnant population was represented in the replies. Survey studies in this setting are necessary but scarce. The study design included qualitative input by allowing free text comments, which revealed that some issues were not addressed in the national guidelines. A limitation is the study’s retrospective design, including questions on the management of pregnancy dating from 1997. Pregnancy dating in clinical practice may differ from the replies provided by the one responder who represented each unit. In addition, the nonresponders may have worked in units that differed in some aspects from those of the responders. However, the response rate was high and there were few nonresponders; therefore, we consider these results to have good generalizability.

In this evaluation of adherence to national guidelines, we identified the existence of unwritten or informal guidelines. This could be discussed during guideline revisions to ensure consensus on evidence-based guidelines to improve clinical implementation. Our findings highlight the importance of an effort to improve the implementation of guidelines in general, which ideally include a multilevel approach involving interventions between educational, practical, and policy-making areas [22]. Local opinion leaders can have a large effect on the degree of implementation [4].

The observed two definitions of gestational age at EDD (39 weeks + 6 days or 40 weeks + 0 days, respectively) imply a risk when patients move between counties as 1 day of difference in gestational age could affect the induction of postterm pregnancies or differentiation of miscarriage from extremely preterm delivery [23]. Also, using the same definition would facilitate comparisons in research [24].

Some units would repeat pregnancy dating performed elsewhere if the documentation was inadequate, which is in conflict with the intention to keep ultrasound exposure as low as reasonably possible to avoid adverse side effects [25, 26].

In conclusion, the units reported good adherence to national guidelines, with the exception of early pregnancy dating. The management of discrepancies between methods for pregnancy dating in clinical practice varied widely and should be considered for inclusion in the national guidelines. This study revealed that some units followed written guidelines whereas others changed practice according to unwritten informal guidelines. This indicates a need for regular updates and efforts to improve the implementation of national guidelines. Finally, follow-up of adherence to guidelines is essential and should be used as a marker of high-quality care.

## Data Availability

The complete datasets generated and/or analyzed during the current study are not publicly available to protect the anonymity of the responders but unidentified responses can be made available from the corresponding author on reasonable request.

## Abbreviations

BPD: biparietal diameter
CRL: crown–rump length
EDD: estimated date of delivery
